# Effect of a Structured Nurse-Led Intervention on Cancer-Related Fatigue During Adjuvant External Beam Radiation Therapy for Breast Cancer: A Randomized Controlled Trial

**DOI:** 10.7759/cureus.114099

**Published:** 2026-08-07

**Authors:** Reena Marandi, Kamli Prakash, Vipul Nautiyal, Anmol Mahani, Sanchita Pugazhendi

**Affiliations:** 1 Department of Nursing, Swami Rama Himalayan University, Dehradun, IND; 2 Radiation Oncology, Cancer Research Institute, Himalayan Institute of Medical Sciences, Swami Rama Himalayan University, Dehradun, IND

**Keywords:** adjuvant external beam radiation therapy (ebrt), breast cancer, cancer-related fatigue, nurse-led intervention, oncology nursing, revised piper fatigue scale

## Abstract

Background: Cancer-related fatigue is a prevalent and distressing symptom among patients receiving adjuvant external beam radiation therapy (EBRT) for breast cancer. Persistent cancer-related fatigue adversely affects physical functioning, psychological well-being, quality of life, and treatment adherence. Structured nurse-led interventions have the potential to alleviate cancer-related fatigue and improve supportive care outcomes. Objective: This study aimed to evaluate the effectiveness of a structured nurse-led intervention compared with usual care in reducing cancer-related fatigue among patients with breast cancer undergoing adjuvant EBRT over a six-month follow-up period. Materials and methods: Eighty-eight women with breast cancer receiving adjuvant EBRT participated in a randomized controlled trial at the Cancer Research Institute, Swami Rama Himalayan University. The Sequentially Numbered Opaque Sealed Envelope (SNOSE) approach was used to randomly assign participants to a control group (n = 44) and an experimental group (n = 44). While the control group received only usual care, the experimental group also received a structured nurse-led intervention. Cancer-related fatigue was assessed using the Revised Piper Fatigue Scale at baseline and at 11 days, 21 days, and one, three, and six months after adjuvant EBRT. Results: The sociodemographic characteristics, clinical variables, and baseline cancer-related fatigue scores were similar between the experimental and control groups. Compared with the control group, the experimental group showed significantly lower cancer-related fatigue scores at 11 days (p = 0.01), 21 days of adjuvant EBRT (p = 0.01), and at one, three, and six months following completion of adjuvant EBRT (all p = 0.001). Repeated within-group analysis also demonstrated a significantly greater reduction in cancer-related fatigue among participants in the experimental group (F = 142.36, p < 0.001) than in the control group. Significant reductions were also observed across cancer-related fatigue domains, for example, behavioral, affective, sensory, and cognitive/mood. Conclusions: The structured nurse-led intervention significantly reduced cancer-related fatigue across all domains among patients undergoing adjuvant EBRT for breast cancer, supporting its incorporation into usual oncology nursing practice. Oncology nurses should integrate this nurse-led intervention into usual radiation care for patients with breast cancer undergoing adjuvant EBRT to reduce their cancer-related fatigue symptoms and improve outcomes.

## Introduction

Breast cancer is the most commonly diagnosed malignancy among women worldwide and remains one of the major contributors to cancer-related illness and death. Advances in screening, diagnosis, and treatment, including multimodal strategies that combine surgery, chemotherapy, radiation therapy, hormonal therapy, and targeted therapy, have substantially improved survival rates; however, treatment-related symptoms continue to negatively affect patients’ quality of life and overall functional status [1].

In the treatment of breast cancer, radiation therapy, specifically external beam radiation therapy (EBRT), is crucial for reducing the risk of local recurrence and improving patient survival. Despite its therapeutic benefits, one of the most common and distressing side effects of EBRT is cancer-related fatigue. Cancer-related fatigue is a multifactorial condition characterized primarily by persistent physical, emotional, and mental exhaustion; this fatigue is disproportionate to recent exertion and is not relieved by rest. Among patients undergoing cancer treatment, cancer-related fatigue severely affects daily activities, emotional well-being, and health-related quality of life [2].

Approximately 35% or more of patients experience cancer-related fatigue during treatment and recovery; it is a common and distressing symptom among women with breast cancer. It significantly impacts the patient's recovery process, quality of life, and physical functioning; furthermore, it may also be linked to long-term survival outcomes. Fatigue has multiple causes that are not yet fully understood, involving medical, psychological, treatment-related, and disease-related factors. Research indicates that fatigue is often most severe during therapy and in the initial six months following treatment completion. This highlights the multifactorial nature of cancer-related fatigue and the need for continued investigation [3].

The causes of breast cancer-related fatigue are complex and not fully understood. However, several molecular factors such as increased oxidative stress, mitochondrial dysfunction, and an imbalance of inflammatory cytokines have been identified as potential causes of the persistent fatigue experienced before and after cancer treatment. Furthermore, while improved treatment modalities such as hypofractionated radiation and reduced chemotherapy exposure have altered the patient experience, fatigue remains a significant and unresolved clinical issue [4].

Furthermore, there is a clear link between cancer-related fatigue and impairments in physical functioning, psychological distress, and cognitive dysfunction; all of these diminish the health-related quality of life for breast cancer survivors. This increases the burden of the disease, as it affects patients' ability to perform daily activities, fulfill social roles, and adhere to treatment plans [5].

Non-pharmacological approaches to managing cancer-related fatigue are gaining increasing popularity. Although the optimal type and intensity of activity have not yet been determined, exercise-based rehabilitation programs have shown promising results in reducing fatigue during adjuvant treatment [6].

Furthermore, it has been observed that structured rehabilitation programs improve functional outcomes and fatigue levels in breast cancer patients, thereby reinforcing their role in comprehensive cancer care. New supportive care techniques such as multimodal and patient-centered interventions have further highlighted the importance of comprehensive symptom management in oncology therapy [7].

Cancer-related fatigue remains a persistent and often undertreated issue during cancer treatment, despite substantial evidence supporting non-pharmacological interventions. Nurses play a crucial role in providing essential care, information, counseling, and symptom monitoring to patients. Nurse-led interventions can be an effective approach to managing fatigue; these programs may incorporate relaxation techniques, diaphragmatic breathing exercises, physical activity, skincare advice, self-care counseling, and dietary guidance [8-12].

In view of these findings, the present study aimed to determine the effectiveness of a structured nurse-led intervention on cancer-related fatigue in patients with breast cancer receiving adjuvant EBRT.

## Materials and methods

A randomized controlled trial was undertaken to determine the effectiveness of a structured nurse-led intervention in alleviating cancer-related fatigue symptoms among patients with breast cancer undergoing adjuvant EBRT. The study was carried out between March 2024 and September 2025 at the Cancer Research Institute, a unit of Himalayan Hospital, Swami Rama Himalayan University, Dehradun, Uttarakhand, India.

The study population consisted of patients diagnosed with breast cancer undergoing adjuvant EBRT in the radiation oncology department of the hospital. Patients aged 18-60 years, who had completed primary treatment, including surgery and chemotherapy, and were willing to participate, were included. Patients receiving palliative radiation, those with neurological disorders, and patients with distant metastases were excluded from the study.

The sample size was estimated through power analysis based on previously published studies evaluating cancer-related fatigue among patients with breast cancer undergoing radiation therapy [9-11]. The calculation was performed using the reported mean and standard deviation of fatigue scores from the reference studies, assuming an 80% power and a 5% level of significance (95% confidence level). After accounting for an anticipated 10% dropout rate, the required sample size was 88 participants, with 44 participants allocated to each of the experimental and control groups. Eligible participants were screened according to the predefined eligibility criteria following ethical approval and trial registration.

Cancer-related fatigue was assessed using the Revised Piper Fatigue Scale (RPFS) [13] before initiation of adjuvant EBRT (Day 0). The RPFS is a validated 22-item instrument measuring behavioral, affective, sensory, and cognitive/mood dimensions of fatigue on a 0-10 scale, with higher scores indicating greater fatigue. The tool demonstrated good reliability using a test-retest method, with calculated r = 0.832.

The primary outcome was the total RPFS score. Secondary outcomes included the behavioral, affective, sensory, and cognitive/mood domain scores. The study hypothesized that the structured nurse-led intervention would significantly reduce cancer-related fatigue compared with usual care.

Participants fulfilling the study eligibility criteria were recruited and randomly allocated to the experimental or control group using a chit-based randomization method with allocation concealment through Sequentially Numbered Opaque Sealed Envelopes (SNOSE). The envelopes were sequentially numbered, opaque, sealed, and opened only after participant enrollment to ensure allocation concealment. Written informed consent was obtained before enrolment. Due to the nature of the intervention, blinding of participants and the intervention provider was not feasible. Demographic and clinical data were collected from all participants. Baseline cancer-related fatigue was assessed using the RPFS before initiation of adjuvant EBRT (Day 0). The structured nurse-led intervention was delivered by the researcher to participants in the experimental group for one hour before each adjuvant EBRT session over 20 consecutive treatment days. On Day 1, participants received individual training through demonstration and discussion on diaphragmatic breathing, systematic relaxation, joint and gland exercises, alternate nostril breathing (Nadi Shodhana), skin and self-care, and dietary guidelines. From Days 2 to 20, participants performed supervised relaxation exercises daily before EBRT. Education and reinforcement regarding dietary practices and skin care were provided on alternate days throughout the intervention period, along with regular feedback on home practice.

Participants in the control group received usual care, including information regarding adjuvant EBRT, side effects, medication, skin care, dietary advice, and personal care as per institutional protocols. Post-test cancer-related fatigue was assessed in both groups on days 11 and 21 of adjuvant EBRT and at one, three, and six months after completion of adjuvant EBRT. Intervention fidelity was ensured through standardized protocols, supervision, participant booklets, and regular monitoring by the researcher.

After data entry into Excel (Microsoft Corp., Redmond, WA, USA), statistical analyses were performed using SPSS Statistics version 26.0 (IBM Corp. Released 2019. IBM SPSS Statistics for Windows, Version 26.0. Armonk, NY). Descriptive measures such as frequency, percentage, mean, and standard deviation were calculated to describe the participants' sociodemographic and clinical variables. Baseline group comparability was assessed using Fisher’s exact test. The Mann-Whitney U test was used for between-group comparisons, while the Friedman test was used for within-group repeated-measures analysis. Statistical significance was established at p < 0.05, with p < 0.01 denoting a highly significant difference.

The study was registered with the Clinical Trials Registry-India (CTRI) (CTRI/2023/05/053164; registered on 26/05/2023; prospectively registered). Ethical approval was obtained from the Institutional Ethics Committee of Swami Rama Himalayan University (approval number: ECR/483/INST/UK-2013/RR-2016). Administrative permission was obtained from the concerned authorities. Prior to participation, written informed consent was obtained from all participants. Confidentiality and anonymity were maintained throughout the research, and participation was entirely voluntary, with the freedom to withdraw from the study at any time.

## Results

**Figure 1:**
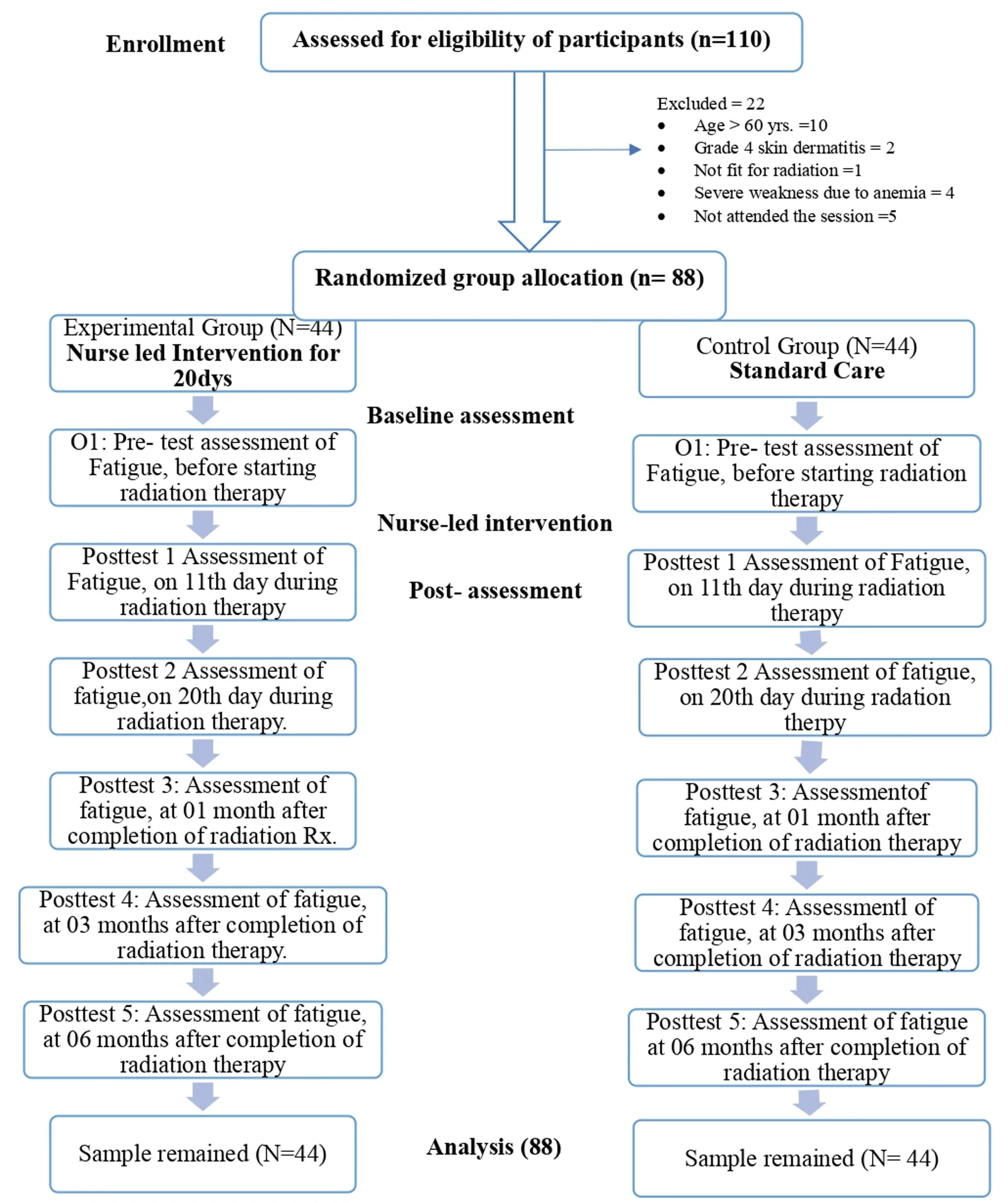
CONSORT flow diagram illustrating participant recruitment, randomization, allocation, follow-up, and analysis

A total of 110 participants were assessed for eligibility, of whom 88 met the inclusion criteria and were randomly allocated to the experimental (n = 44) and control (n = 44) groups. Both groups completed the planned follow-up assessments, and all participants were included in the final analysis (Figure 1).

The sociodemographic and clinical characteristics of participants were comparable between the experimental and control groups. Baseline comparisons revealed similar characteristics between the groups in age, education, religion, family type, dietary pattern, financial status, occupation, living arrangement, area of residence, smoking history, family history of breast cancer, menstrual history, disease stage, surgery type, pathological stage, pain level, radiation dose, or hemoglobin level (p > 0.05). These findings confirmed baseline homogeneity between groups (Table 1).

**Table 1:** Sociodemographic characteristics of patients with breast cancer undergoing adjuvant EBRT EBRT: external beam radiation therapy

Sociodemographic variables		Control group (n = 44), frequency (%)	Experimental group (n = 44), frequency (%)	Fisher's exact test	p-value
Age (years)		47.30 ± 7.99	44.52 ± 10.08	1.43	0.156
Educational status	Graduate and above	13 (29.5%)	14 (31.8%)	2.249	0.522
	No formal education	9 (20.5%)	14 (31.8%)		
	Primary level education	19 (43.2%)	13 (29.5%)		
	Secondary level education	3 (6.8%)	3 (6.8%)		
Religion	Hindu	36 (81.8%)	37 (84.1%)	2.973	0.272
	Muslim	5 (11.4%)	7 (15.9%)		
	Sikh	3 (6.8%)	0 (0.0%)		
Type of family	Joint	17 (38.6%)	18 (40.9%)	0.047	1.000
	Nuclear	27 (61.4%)	26 (59.1%)		
Dietary pattern	Non-vegetarian	18 (40.9%)	22 (50.0%)	0.733	0.521
	Vegetarian	26 (59.1%)	22 (50.0%)		
Financial situation	<50,000	24 (54.5%)	26 (59.1%)	0.185	0.830
	>50,000	20 (45.5%)	18 (40.9%)		
Occupation	Government employee	15 (34.1%)	11 (25.0%)	1.481	0.794
	Homemaker	6 (13.6%)	9 (20.5%)		
	Private employee	22 (50.0%)	23 (52.3%)		
	Self-employment	1 (2.3%)	1 (2.3%)		
Living with	Alone	0 (0.0%)	1 (2.3%)	3.738	0.465
	Only with children	2 (4.5%)	0 (0.0%)		
	Other family members	8 (18.2%)	12 (27.3%)		
	Spouse	1 (2.3%)	1 (2.3%)		
	Spouse and children	33 (75.0%)	30 (68.2%)		
Area of living	Rural	17 (38.6%)	16 (36.4%)	0.048	1.000
	Urban	27 (61.4%)	28 (63.6%)		
Operating expense	Empanelled	28 (63.6%)	30 (68.2%)	0.202	0.822
	Self-payment	16 (36.4%)	14 (31.8%)		
Past smoking history	No	44 (100.0%)	42 (95.5%)	2.047	0.494
	Yes	0 (0.0%)	2 (4.5%)		
Family history of breast cancer	No	44 (100.0%)	41 (93.2%)	3.106	0.241
	Yes	0 (0.0%)	3 (6.8%)		
Menstrual history	Post-menopause	21 (47.7%)	12 (27.3%)	4.327	0.115
	Regular	6 (13.6%)	6 (13.6%)		
	Treatment-induced menopause	17 (38.6%)	26 (59.1%)		

There was no significant difference in cancer-related fatigue scores between the groups at baseline, indicating that the groups were comparable. During adjuvant EBRT, a significant difference (p = 0.001) was observed on day 11, whereas no significant difference was noted on day 21. From the one-month mark onwards, cancer-related fatigue scores were significantly reduced among participants in the experimental group compared with those in the control group, and this effect persisted at three and six months (p < 0.001); the lowest cancer-related fatigue scores in the experimental group were observed at the six-month follow-up. Within-group analysis (Friedman test) revealed significant changes over time in both groups (p < 0.001), indicating fluctuations in cancer-related fatigue during the follow-up period. Overall, participants in the experimental group demonstrated significantly lower cancer-related fatigue scores than the control group at one, three, and six months after adjuvant EBRT (p < 0.001). However, no significant difference was observed at baseline or Day 20 (Table 2).

**Table 2:** Comparison of total cancer-related fatigue scores of patients with breast cancer undergoing adjuvant EBRT between experimental and control groups ** Highly statistically significant (p < 0.01) EBRT: external beam radiation therapy, IQR: interquartile range

Assessment	Experimental group (n = 44), median (IQR)	Control group (n = 44), median (IQR)	Mann-Whitney U test	p-value
Baseline	4.40 (4.00-5.20)	4.50 (4.10-5.10)	944.500	0.844
11 days of radiation therapy	4.50 (4.50-7.30)	5.50 (5.40-7.50)	565.500	0.001**
21 days of radiation therapy	5.00 (4.80-7.70)	5.60 (5.50-7.70)	775.000	0.105
1 month after radiation therapy	4.60 (4.30-5.10)	6.80 (5.20-7.60)	171.500	0.001**
3 months after radiation therapy	3.70 (3.50-4.00)	6.90 (6.50-7.20)	0.000	0.001**
6 months after radiation therapy	2.80 (2.80-3.10)	4.50 (4.00-5.20)	2.500	0.001**
Friedman χ² within-group p-value	142.36 0.001**	98.45 0.001**	-	-

**Figure 2:**
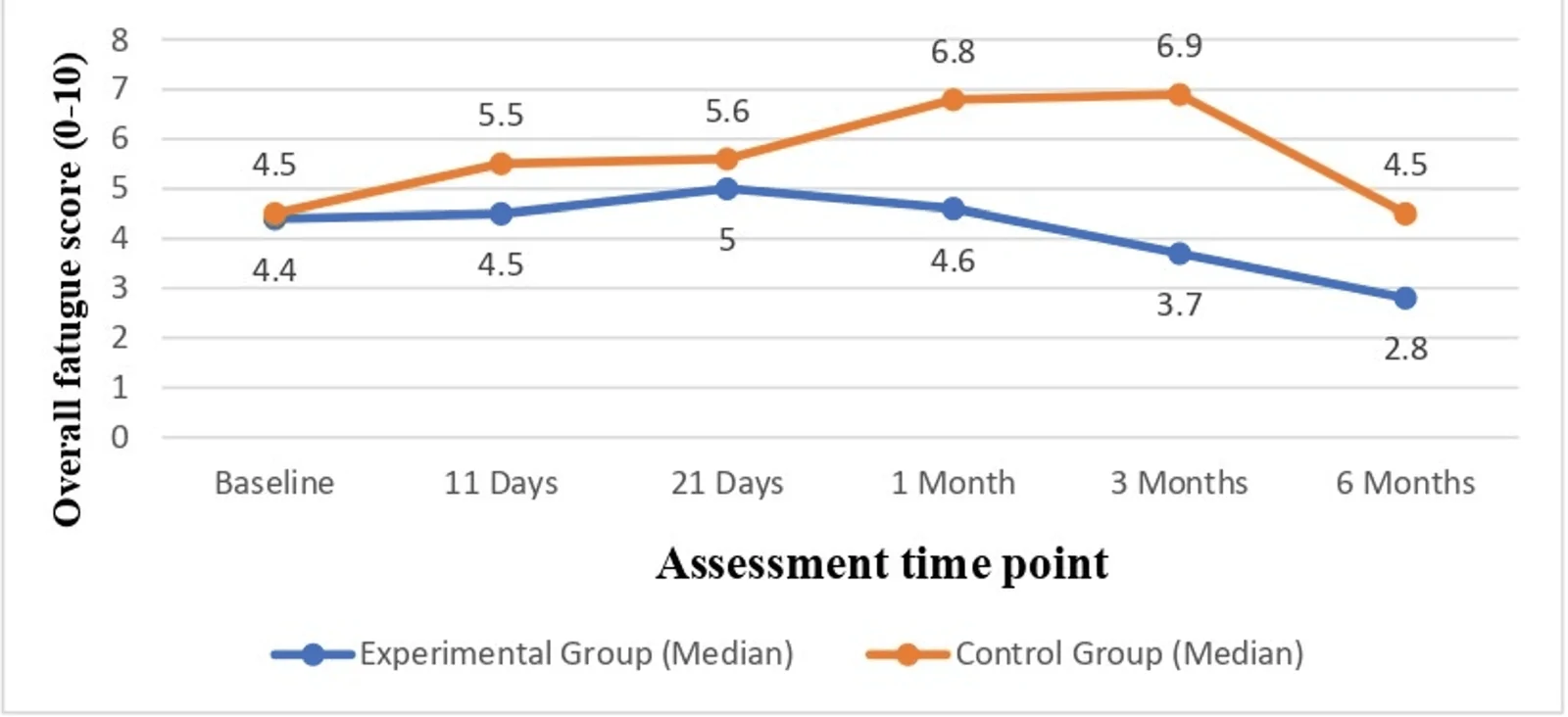
Comparison of overall median cancer-related fatigue scores between the experimental and control groups over time

The line graph illustrates the pattern of change in cancer-related fatigue scores over time in both groups. Lower cancer-related fatigue scores were observed in the experimental group than in the control group during the follow-up assessments (Figure 2).

The comparison of cancer-related fatigue domains (behavioral/severity, affective, sensory, and cognitive/mood) between the experimental and control groups across six assessment time points was performed using the Mann-Whitney U test. In contrast, within-group changes over time were assessed using the Friedman test. At baseline, both groups demonstrated comparable scores across all cancer-related fatigue domains, with no statistically significant between-group differences (p > 0.05). During adjuvant EBRT, significant between-group differences were observed in the behavioral/severity and affective domains at Day 11 (p = 0.001 and p < 0.001, respectively). In contrast, no significant differences were observed at Day 20, except for a borderline difference in the sensory domain (p = 0.053) (Table 3).

**Table 3:** Comparison of cancer-related fatigue domains of patients with breast cancer receiving adjuvant EBRT within and between the experimental and control groups * Statistically significant (p < 0.05), ** highly statistically significant (p < 0.01) EBRT: external beam radiation therapy, IQR: interquartile range

Fatigue domain	Assessment	Experimental group (n = 44), median (IQR)	Control group (n = 44), median (IQR)	Mann-Whitney U test	p-value
Behavioral/severity	Baseline	30.50 (28.00-32.75)	31.00 (24.00-37.00)	944.500	0.844
	11 days of adjuvant EBRT	40.00 (38.00-42.00)	30.00 (30.00-42.00)	565.500	0.001**
	21 days of adjuvant EBRT	41.00 (38.25-42.75)	35.50 (30.00-45.00)	775.000	0.105
	1 month after adjuvant EBRT	40.00 (35.25-42.00)	31.50 (30.00-35.00)	171.500	<0.001**
	3 months after adjuvant EBRT	36.00 (36.00-37.00)	21.00 (21.00-24.00)	0.000	<0.001**
	6 months after adjuvant EBRT	28.00 (26.00-29.00)	17.00 (17.00-18.00)	2.500	<0.001**
	Friedman χ² (within-group), p-value	161.261, <0.001	157.244, <0.001	-	-
Affective fatigue	Baseline	20.00 (18.00-22.00)	20.00 (17.25-22.75)	918.000	0.673
	11 days of adjuvant EBRT	34.00 (32.50-38.75)	25.00 (25.00-36.00)	464.500	<0.001**
	21 days of adjuvant EBRT	37.00 (33.25-40.00)	32.50 (24.25-40.00)	749.000	0.065
	1 month after adjuvant EBRT	38.00 (32.00-41.00)	20.00 (19.00-22.00)	0.000	<0.001**
	3 months after adjuvant EBRT	34.00 (34.00-35.00)	16.00 (13.25-17.00)	0.000	<0.001**
	6 months after adjuvant EBRT	23.00 (20.00-25.00)	5.00 (5.00-10.00)	0.000	<0.001**
	Friedman χ² (within-group), p-value	161.137, <0.001	131.703, <0.001	-	-
Sensory fatigue	Baseline	21.00 (19.00-23.00)	21.50 (19.25-23.75)	855.500	0.345
	11 days of adjuvant EBRT	23.00 (23.00-33.00)	21.00 (21.00-32.00)	646.500	0.007*
	21 days of adjuvant EBRT	26.00 (23.00-33.75)	30.00 (24.25-40.00)	737.500	0.053
	1 month after adjuvant EBRT	30.00 (25.00-34.75)	20.50 (19.00-21.00)	114.500	<0.001**
	3 months after adjuvant EBRT	34.00 (32.00-34.00)	21.00 (19.00-21.00)	0.000	<0.001**
	6 months after adjuvant EBRT	23.50 (21.00-29.75)	18.00 (17.00-18.00)	56.000	<0.001**
	Friedman χ² (within-group), p-value	148.553, <0.001	128.247, <0.001	-	-
Cognitive/mood fatigue	Baseline	24.00 (22.00-26.00)	26.00 (23.00-28.75)	784.500	0.124
	11 days of adjuvant EBRT	30.50 (26.00-48.00)	24.50 (22.00-45.00)	913.000	0.637
	21 days of adjuvant EBRT	41.00 (29.25-48.00)	35.50 (28.00-48.00)	794.000	0.142
	1 month after adjuvant EBRT	44.50 (39.00-48.00)	28.50 (22.25-33.00)	358.500	<0.001**
	3 months after adjuvant EBRT	27.00 (25.00-29.00)	19.00 (18.00-22.75)	0.000	<0.001**
	6 months after adjuvant EBRT	27.00 (25.00-29.00)	22.00 (21.00-24.00)	201.500	<0.001**
	Friedman χ² (within-group), p-value	107.528, <0.001	129.074, <0.001	-	-

From one month onward, statistically significant between-group differences were observed across all cancer-related fatigue domains (p ≤ 0.001), and these differences were maintained at the three- and six-month follow-up assessments. Within-group analysis using the Friedman test demonstrated significant changes over time in all cancer-related fatigue domains in both groups (p < 0.001). Overall, cancer-related fatigue domain scores changed significantly over time in both groups (Friedman test, p < 0.001). Significant between-group differences were observed from one month onward across all domains (p ≤ 0.001) (Table 3).

Overall, the findings demonstrated that both groups experienced significant changes in cancer-related fatigue over time (Friedman test, p < 0.001). Baseline assessment confirmed that the experimental and control groups were comparable in terms of overall cancer-related fatigue, fatigue domains, and sociodemographic characteristics (p > 0.05). Although only limited between-group differences were observed during adjuvant EBRT, statistically significant differences favoring the experimental group were evident from one month after completion of adjuvant EBRT. They were maintained at the three- and six-month follow-up assessments (p ≤ 0.001). These findings indicate that participants who received the structured nurse-led intervention demonstrated greater improvement in cancer-related fatigue than those receiving usual care over the follow-up period, supporting the effectiveness of the intervention.

## Discussion

The current study assessed whether a structured nurse-led intervention consisting of relaxation techniques could reduce cancer-related fatigue symptoms in patients with breast cancer undergoing adjuvant EBRT. The intervention group showed sustained, statistically significant improvement in overall cancer-related fatigue scores compared with the control group across the six-month follow-up, demonstrating the effectiveness of the structured nurse-led intervention. Treatment-related cancer-related fatigue is a frequent and burdensome consequence of adjuvant EBRT that can adversely affect patients' physical, emotional, cognitive, and social well-being. The present findings suggest that integrating structured nurse-led supportive care into usual oncology practice may substantially improve cancer-related fatigue management and overall patient well-being.

The intervention resulted in significant reductions in the behavioral/severity dimension of cancer-related fatigue, indicating enhanced physical functioning and greater ability to perform daily activities. These improvements may be attributed to the relaxation-based components of the intervention, including diaphragmatic breathing, alternate nostril breathing (pranayama), systematic relaxation, and joint and gland exercises, which improve oxygenation, physical endurance, and energy conservation during treatment. These findings are consistent with those of Alcântara-Silva et al., who reported that pranayama and breathing exercises significantly reduced fatigue and improved sleep quality in patients receiving radiation therapy [14]. Similarly, André et al. and Medeiros Torres et al. demonstrated that structured exercise-based supportive interventions effectively improve physical functioning and alleviate cancer-related fatigue during treatment [15,16].

Significant improvement was also observed in the affective domain of cancer-related fatigue, suggesting better emotional well-being and psychological adaptation among patients receiving the intervention. Regular counseling, relaxation techniques, and continuous nurse-patient interaction may have enhanced coping abilities and reduced treatment-related emotional distress. These findings are supported by Trisyani et al., who reported that psychosocial nursing interventions reduce fatigue by strengthening coping mechanisms and decreasing emotional distress [17]. Likewise, Xu et al. found that structured nursing interventions improve symptom management and emotional well-being among women with breast cancer [18].

The nurse-led intervention also significantly reduced sensory fatigue, reflected by improvements in weakness, tiredness, and lack of energy. The combined effects of exercise, skin care education, self-care practices, and dietary counseling may have contributed to better symptom control and physical comfort during adjuvant EBRT. These findings agree with Abreu et al., who emphasized the importance of nursing interventions in managing radiation therapy-related symptoms; Oppermann et al., who demonstrated the benefits of nutritional interventions during radiation therapy; and Zuo et al., who reported that structured nursing interventions incorporating lifestyle and dietary guidance effectively reduce cancer-related fatigue [19-21].

Furthermore, significant improvement was observed in the cognitive/mood domain of fatigue, indicating better concentration, mental alertness, emotional stability, and psychological functioning among patients receiving the intervention. These improvements may be explained by enhanced symptom control, increased self-efficacy, and reduced treatment-related stress achieved through structured education and continuous nursing support. Similar findings have been reported by Manajreh et al. and Nayak et al., who highlighted the importance of nursing care in managing radiation therapy-related fatigue and promoting patient self-management [22,23]. Alcântara-Silva et al. and Courtier et al. also demonstrated that relaxation-based complementary therapies improve psychological well-being and reduce treatment-related fatigue [14,24].

Overall, the findings of the present study supported the integration of structured nurse-led interventions into usual oncology care for patients undergoing adjuvant EBRT. A multimodal approach combining breathing exercises, relaxation techniques, self-care education, skin care, and dietary counseling effectively reduced overall and multidimensional cancer-related fatigue, thereby improving patients' physical, emotional, sensory, and cognitive well-being during and after treatment.

Strengths of the study

The strengths of this study include its randomized controlled trial design, prospective registration with the CTRI, use of the validated RPFS to comprehensively assess multidimensional cancer-related fatigue, and a six-month follow-up period that enabled evaluation of both short- and long-term intervention effects. Baseline comparability between the experimental and control groups further strengthened the internal validity of the findings. Additionally, all enrolled participants completed the planned six-month follow-up assessments, resulting in complete outcome data for analysis and eliminating loss to follow-up.

Limitations of the study

The study has certain limitations. It was conducted at a single cancer care center with a relatively small sample of participants aged 18-60 years, which may limit the generalizability of the findings. Blinding of participants, the intervention provider, and outcome assessment was not feasible because of the nature of the behavioral intervention, which may have introduced performance and detection bias. Additionally, the intervention was delivered by a single researcher, which may limit the reproducibility of the findings in other settings. Furthermore, effect sizes and confidence intervals were not reported, which may limit the interpretation of the magnitude and precision of the observed intervention effects.

## Conclusions

The study findings support the use of a structured nurse-led intervention to improve cancer-related fatigue outcomes in patients with breast cancer undergoing adjuvant EBRT. Significant improvements were observed across all dimensions of cancer-related fatigue, including behavioral/severity, affective, sensory, and cognitive/mood domains. The beneficial effects were sustained up to six months following completion of treatment. These findings support integrating nurse-led cancer-related fatigue management strategies into usual oncology care to improve patient outcomes and quality of life.
